# Atrazine degradation through PEI-copper nanoparticles deposited onto montmorillonite and sand

**DOI:** 10.1038/s41598-017-01429-5

**Published:** 2017-05-03

**Authors:** Sethu Kalidhasan, Ishai Dror, Brian Berkowitz

**Affiliations:** Department of Earth and Planetary Sciences, Weizmann Institute of Science, Rehovot, 7610001 Israel

## Abstract

We present the synthesis of new composite materials based on copper nanoparticles (Cu NPs) deposited onto montmorillonite (MK10) and quartz sand, for degradation of atrazine, in the context of an advanced oxidation process (AOP). The synthesis involves a first step in which polyethylenimine (PEI) capped Cu NPs (PEI_Cu NPs) are prepared, and then deposited onto, separately, MK10 and sand, through a solvent impregnation method. The resulting products are characterized in detail; the copper is found to exist as a mixture of copper (I, II) oxide. The degradation of atrazine follows a second-order kinetic model with constant values of K_2_ = 1.7957 g mg^−1^ min^−1^ for MK10_PEI_Cu NPs and K_2_ = 0.8133 g mg^−1^ min^−1^ for sand_PEI_Cu NPs. The reaction rate is linked to Cu_2_O and CuO redox-active species within the layers, pores and surface of the host materials. A degradation mechanism is found with application of these composite materials in the presence of H_2_O_2_; adsorption occurs in the absence of H_2_O_2_. In contrast, the unmodified MK10 and sand exhibit adsorption in both of the above reaction conditions. Finally, the stability of the Cu NPs following degradation is evaluated, and no significant amount of copper leaching is found.

## Introduction

Residues of the pesticide atrazine [2-chloro-4-ethylamino-6-isopropylamino-s-triazine] have been found in many water resources around the world. Atrazine has low solubility in water (1.6 × 10^−4^ M at 20 °C) and is considered relatively stable and persistent in aqueous environments and soils. Currently, atrazine is one of the most toxic, heavily used herbicides in North America^1, 2^, while in Europe its use was banned more than a decade ago^3^. The maximum allowed concentration of atrazine in drinking water in the USA is 3 ppb, and, suitable methods for its detoxification and removal from water are needed. Several materials and methods have been suggested for the removal of atrazine^4, 5^. Reported studies focus on reverse osmosis (RO)^6^, nanofiltration (NF)^7^, physicochemical methods such as extraction and adsorption^8^, and degradation based on visible or ultraviolet radiation, heat, microwave and ultrasound^9–14^. Each method has advantages and drawbacks. For example, some methods are expensive or suffer from membrane fouling due to accumulation of colloidal particles.

In general, the most commonly used method for atrazine elimination from water involves adsorption by various materials including activated carbon^15^, porous materials^16^, biowastes/biomass^17–19^, nano metal oxide^20–23^, and clays^24, 25^. On the other hand, few of the above-mentioned materials eliminate atrazine by chemical or photocatalytic degradation methods^22, 23, 26, 27^. Among these materials, combinations of clays and metal oxides have received attention, but only limited studies exist on catalytic performance.

Clays and sand are natural, abundant, inexpensive and environmentally friendly materials. The net charge of montmorillonite K10 (MK10) is purely dependent on its substitution by, e.g., surfactants or metal cations; this ion exchange ability of montmorillonite makes it suitable for embedding ionic species via ion exchange method^28^, resulting in the formation of efficient heterogeneous Fenton catalysts. Wet impregnation and incipient wetness impregnation are additional methods for introducing small molecules into porous and layered materials^29, 30^.

Application of metal oxides loaded onto sand and clay for heterogeneous catalysis is a promising alternative for decontamination of soils, groundwater, sediments, and industrial effluents. The immobilization of active species on insoluble matrices is useful because it minimizes loss of the metal and facilitates its separation from the reaction mixture, thus enabling reuse of the catalysts. Djeffal *et al*.^31^ used Fe-rich red clay as an heterogeneous Fenton catalyst for the degradation of phenol and tyrosol in an aqueous medium, observing complete removal of the pollutant and 70% mineralization of the solution after 6 h of contact time. De León *et al*.^32^ employed montmorillonite pillared by iron for the degradation of methylene blue in the presence of UV light, while George *et al*.^33^ examined pillared montmorillonite-supported ferric oxalate as an heterogeneous photo Fenton catalyst for the degradation of amoxicillin in an aqueous medium. A similar montmorillonite catalyst intercalated with trinuclear iron clusters was used for Fenton photodegradation of phenol by Zhang *et al*.^34^ Muthuvel *et al*.^35^ synthesized a Fe-encapsulated montmorillonite catalyst for the degradation of acid yellow 17 dye and observed high removal efficiency even at neutral pH. A montmorillonite nanocomposite pillared by mesoporous iron-modified Al_2_O_3_ nanoparticles was found to be an effective Fenton photocatalyst for the degradation of acid blue and reactive blue^36^. The iron-containing ball clay was prepared for the oxidative removal of reactive blue 4 and acid red 1 from aqueous solution^37^. Similarly, catalytic degradation of pollutants using a copper oxide loaded sand matrix has been reported^38^. Different Fenton type catalysts have been applied for the degradation of atrazine through both chemical and photochemical degradation^23, 26^. Copper-based Fenton type catalysts, in particular, are simple, effective, and cheaper than most other metal catalysts^39–42^.

A variety of reagents have been used to synthesize copper structures with different sizes^43–50^. Among these, polymer-capped or stabilized materials, involving polymer-containing amine groups such as branched polyethylenimine (bPEI), have received attention; bPEI has important subgroups such as primary, secondary and tertiary amino groups. The amine groups have the ability to complex with copper, which makes these compounds attractive to synthesize copper nanoparticles that will form stable suspensions in aqueous solutions. The advantage of using amino-protecting groups is their higher stability under different conditions, relative to other protective functional group polymers^51–54^.

To date, chemical degradation of atrazine, as a representative persistent water pollutant, using polymer-capped metal catalysts deposited on silicate matrices such as clay and sand has not been explored in detail. This paper describes the preparation and characterization of water-phase-synthesized PEI capped copper nanoparticles (PEI_Cu NPs) and their assimilation onto layered montmorillonite (MK10_PEI_Cu NPs) and sand (sand_PEI_Cu NPs), in separate treatments, through a solvent impregnation method. We also report a study on the catalytic activity of these materials in the degradation of atrazine by H_2_O_2_ in aqueous solution.

## Results and Discussion

### Catalyst Characterization

The properties of the modified MK10 and sand are discussed first; we examine the amount of deposited copper, functional group changes, and material composition and surface morphology. Atrazine degradation is then reported. Initially, we synthesized aqueous suspensions of PEI_Cu NPs. The average particle size of PEI_Cu NPs measured by DLS was 55 nm; the zeta potential of the PEI_Cu NP suspension was 33.4 mV and the suspension was stable for more than one month. The PEI_Cu NPs were deposited onto MK10 and sand as described in the experimental part. Leaching tests by treating each material with 10 mL of 10 wt% nitric acid for 24 h followed by ICP-MS analysis of the supernatant confirmed the deposition of PEI_Cu NPs. The amounts of Cu in modified MK10 and sand were calculated to be 255 mg g^−1^ and 75 mg g^−1^, respectively.

### XRD, XPS and Raman analysis

**Figure 1:**
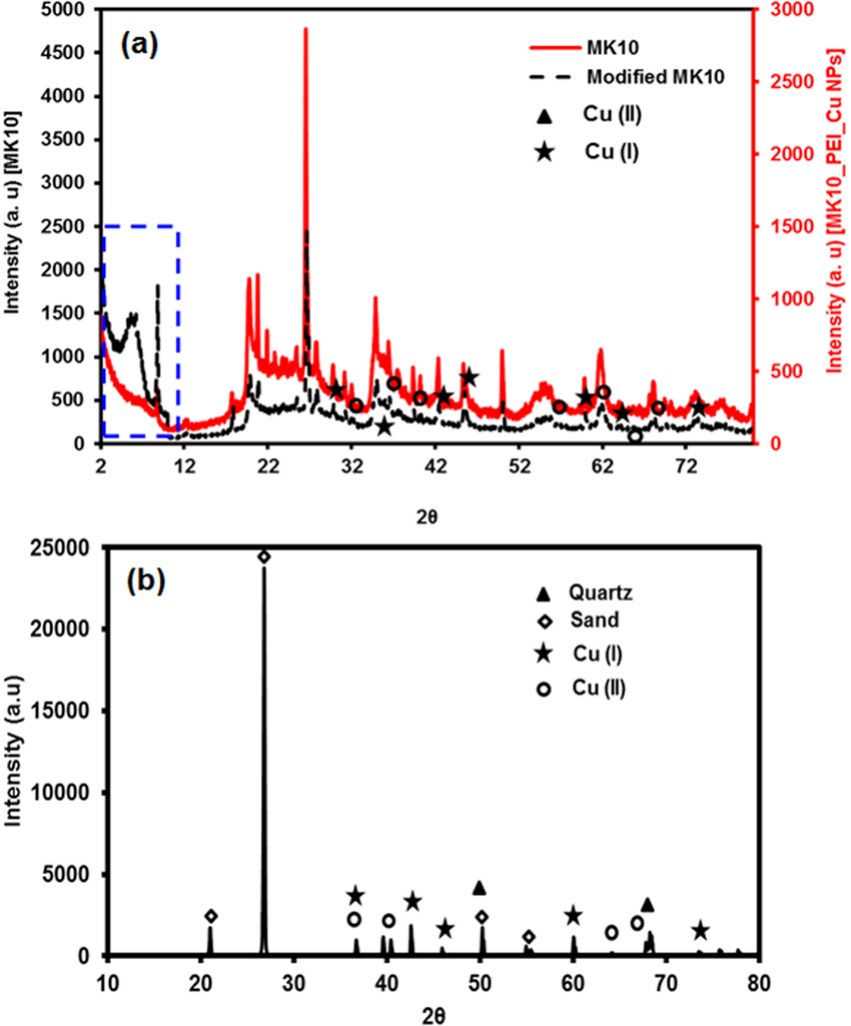
XRD pattern of (**a**) MK10 (before and after the deposition of PEI_Cu NPs); blue dashed box is related to the 001 plane, and (**b**) sand.

The deposition of PEI_Cu NPs onto MK10 and sand was analyzed with powder XRD (X-Ray Diffraction). The XRD patterns (2° to 80° 2θ) for the MK10 control and MK10_PEI_Cu NPs are shown in Fig. 1a, while XRD patterns (10° to 80° 2θ) for the sand_PEI_Cu NPs appear in Fig. 1b. In both cases (Fig. 1a and b), peaks related to Cu_2_O and CuO species are marked with different symbols (star, triangle and circle)^46, 47, 55^. Identifying the peaks associated with copper species within the noisy XRD patterns of MK10 and MK10_PEI_Cu NPs is challenging in some cases. The change in the XRD pattern is very small as only trace amounts of copper were deposited on the clay, while PEI coating further masks reading of the Cu peaks. However, small changes in the XRD patterns of MK10_ PEI_Cu NPs compared to unmodified MK10 show pointed diffraction peaks at 2θ values corresponding to 18.14°, 20.73°, and 22.79°; the crystalline nature of MK10, with a certain degree of exfoliation and inner layer expansion (2θ = 3–9°, marked in blue color box), was reported in several previous publications^56–58^. The resulting non-periodic structure for MK10_PEI_Cu NPs was manifested as a convolution of the higher order 00l reflections. A significant shift in the position of the 001 reflection indicates expansion of the interlayer space, and broadening of the 00l reflections (~2 Å) due to smaller volumes of coherent order among the atoms in the MK10_PEI_Cu NPs. This suggests that ordered structures extended over significantly smaller domains in the intercalated samples as compared to the control (MK10, marked in the blue dashed line box). The shoulder at approximately 2θ = 10° suggests that some of the crystalline domains were altered in the MK10_PEI_Cu NPs and contain interlayer space where PEI is found in sufficient amounts to be detected above background. It is further noted that the existence of different copper species, i.e., Cu°, Cu^+^, and Cu^2+^, was discussed recently^59^, with the XRD pattern of PEI_Cu NPs in suspension in that study being similar to the suspension used for deposition onto MK10 and sand in the present study.

The XPS (X-ray Photoelectron Spectroscopy) surface analysis^60–62^ shows the presence of Cu^2+^ on the surfaces of both MK10_PEI_Cu NPs and sand_PEI_Cu NPs (see Fig. S1 in the Supplementary Information). The MK10_PEI_Cu NPs sample shows weaker peak signals for copper, due to the presence of the non-conducting PEI–polymeric alkyl chain which coats the copper particles; the weaker peak signals are due also to the lower atomic abundance of copper in the sample (0.2 and 0.13%, see Fig. S1c). To overcome this limitation, we used a charge neutralizer during measurements. As a result, all peaks are shifted slightly to lower binding energy (BE) values, compared to the literature. The shift in BE was found to be 1.9 eV for MK10_PEI_Cu NPs and 1.7 eV for sand_PEI_Cu NPs. In addition, use of a charge neutralizer during measurements may change the oxidation state of copper, driving the copper to its higher oxidation state (+2).

Finally, Raman analysis given in Fig. S2 in the Supplementary Information offers additional proof of the presence of both Cu^+^ and Cu^2+^ species, for both MK10_PEI_Cu NPs and sand_PEI_Cu NPs. Samples of pure Cu_2_O and CuO exhibited characteristic Raman bands at 216/420/623 cm^−1^ and 274/318/609 cm^−1^, respectively^63^. The presence of trace copper (I) and copper (II) oxide in MK10_PEI_Cu NPs and sand_PEI_Cu NPs is marked by red colored arrows in Fig. S2 in the Supplementary Information.

### SEM analysis

**Figure 2:**
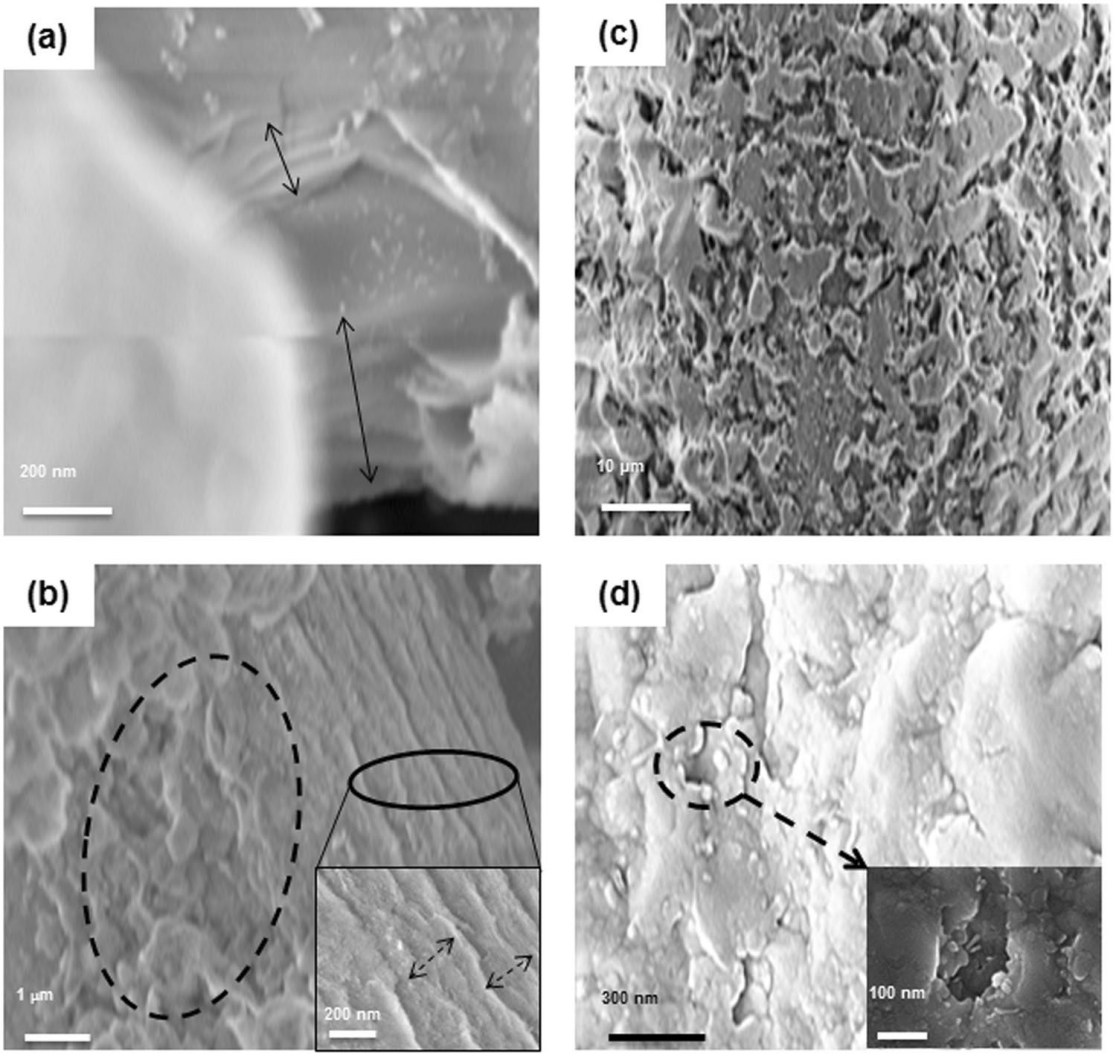
SEM images of (**a**) unmodified MK10; arrows indicate layer structures, (**b**) MK10_PEI_Cu NPs; dotted ellipse show small exfoliation and insert show higher magnification and arrows indicating slight expansion of the layer structure to ~2 Ǻ (**c**) sand, and (**d**) sand_PEI-Cu NPs; dotted ellipses and insert show higher magnification and deposited PEI_Cu NPs.

The deposition of PEI Cu NP on both matrices (sand, MK10) was also confirmed by SEM (Scanning Electron Microscopy) analysis (Fig. 2a–d). Energy-dispersive X-ray spectroscopy (EDS) mapping confirmed copper distribution on the matrix, as shown in Fig. S3 in the Supplementary Information. The unmodified MK10 shows layer structures (Fig. 2a, marked with double-headed arrow). Exfoliation of layer sheets is marked by the dotted circle in Fig. 2b. The insert in Fig. 2b and the double headed arrows show a slightly expanded layer structure of the MK10_PEI_Cu NP matrix. The small exfoliation arises from the deposition of bulkier PEI in PEI_Cu NPs. In sand, the PEI_Cu NPs are deposited into pores/layers of the quartz, and the disc shaped PEI_Cu NPs can be observed (Fig. 2d, marked as a circle with insert SEM image); these features are not found on the unmodified sand (Fig. 2c).

### Thermogravimetric analysis (TGA)

The TGA patterns of both MK10 and sand (unmodified and modified) are given in Fig. S4a–d in the supporting information. In both cases, the initial (low temperature) change shown during the TGA measurements is attributed to loss of low volatile organic compounds and moisture. The significantly different degradation patterns observed between 300 °C and 400 °C in Fig. S4a and b indicate the presence of PEI_Cu NPs in MK10_PEI_Cu NPs. The TGA pattern of PEI alone with MK10 is shown in Fig. S5a,b in the Supplementary Information; PEI has two degradation temperatures, at 330 °C and 370 °C. Generally, the thermal decomposition of copper oxide starts from 300 °C. The remaining small changes evidently arose from variations in MK10 composition^64–66^. We found analogous observations in sand_PEI_Cu NPs for PEI, copper oxide and sand composition.

### FT-IR analysis

**Figure 3:**
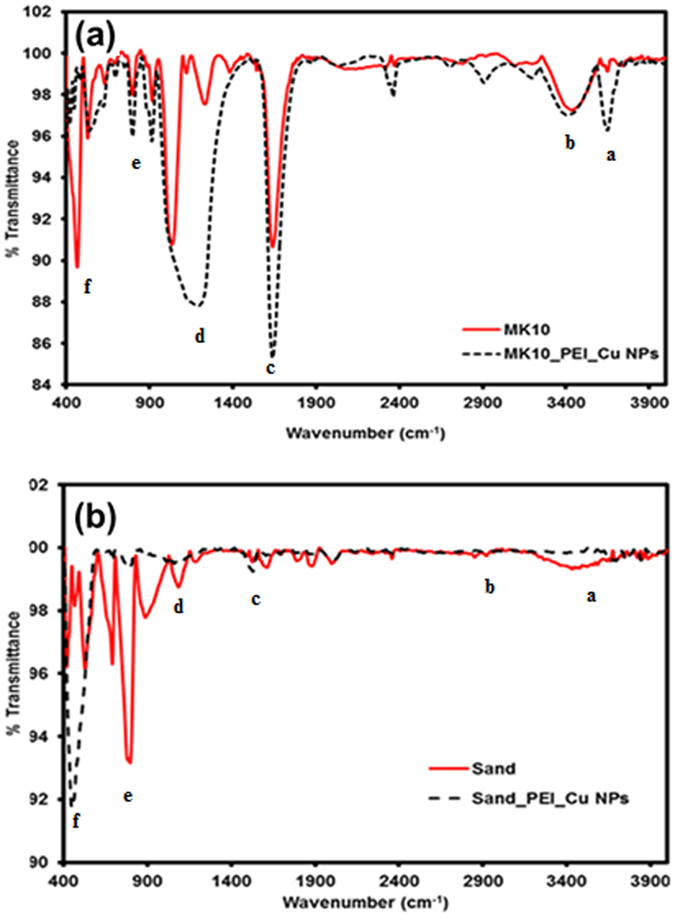
FT-IR spectra of unmodified and modified (**a**) MK10 and (**b**) sand.

The FT-IR spectra of unmodified MK10 and sand were compared to the same materials with PEI_Cu NPs (Fig. 3a,b). Table 1S (see the Supplementary Information) offers a possible interpretation of the FT-IR peak position that corresponds to the significant peak found for MK10_PEI_Cu NPs. The spectrum of the MK10_PEI_Cu NPs shows signals of all constituents of the reactant materials (PEI and Cu), which verifies deposition of PEI_Cu NPs onto the solid matrix. The FT-IR spectra of the matrices (montmorillonite and sand) before and after deposition of PEI-Cu NPs were used to study the deposition of PEI_Cu NPs by comparison of the different peaks and inter-comparisons of relative size within each spectrum. Primarily, the broad and strong band ranging from 3000 to 3800 cm^−1^ can be assigned to overlapping -OH and -NH groups (marked as area “a”). The band at 2840–3000 cm^–1^ denotes the asymmetric and symmetric C-H stretching frequencies of the -CH_2_ group in PEI chains (marked as area “b”). This confirms the presence of amino groups from PEI.

We observe the broader peak in the spectral range 400–800 cm^−1^ relative to the unmodified clay (marked as area “f”). The broadening might be due to the bulkier organic moiety from PEI (CH_2_ rocking, N–H out of plane wagging, C–C bending) and copper oxide (vibrational modes of the Cu–O bond, 479.8 and 585.6 cm^−1^), which may suggest more non-covalent interaction with MK10. Additionally, we observe a change in the peak intensity (Al–Al-OH deformation, 820–920 cm^−1^, marked as area “e”) after modification. The increases in the intensity of the peaks in the spectral range (820–920 cm^−1^, marked as area “e”) after PEI_Cu NP deposition onto MK10 are possibly due to the addition of organic moieties (C–H bending out of plane and C–C skeletonic stretching) from PEI. Likewise, we compared the FT-IR spectrum of the original matrix (montmorillonite and sand, areas “c” and “e”, using them as an internal standard) to the same matrix after the deposition of PEI-Cu NPs.

We also observe a significant increase in peak height and broadening in the spectral range (950–1450 cm^−1^, see Table 1S in the Supplementary Information) for modified MK10, which is due to the amine (C-N-H_n_) peak in non-covalent interaction with neighboring functional group (marked as area “d”). These changes are additional confirmation that PEI_Cu NPs are deposited onto MK10. The strong absorption band at ~1048 cm^−1^ (marked as area “c”) is the uniquely characteristic vibration of Si–O, Si–O–Si in the clay lattice, CH_3_ rocking, overlap of C–C stretching, CH_2_ twisting, C–N stretching, CH_2_ rocking and skeletonic stretching. Moreover, the increased peak intensity at ~1630 cm^−1^ is also assigned to the O–H deformation of entrapped water in clay and N–H bending from PEI, indicating the deposition of PEI_Cu NPs (marked as area “c”).

For sand_PEI_Cu NPs, we observe very weak peak changes in the spectrum when comparing the unmodified and modified sand (Fig. 3b). Nevertheless, we notice that significantly smaller peak changes (similar peak positions, marked as area “a–f”) are due to the smaller amount of PEI_Cu NPs deposited onto sand than onto MK10 (as confirmed by the elemental mapping; see Fig. S3 in the Supplementary Information). Further, weak peak changes and broadening are due possibly to overlapping of the other spectral peaks of copper oxide, hydroxyl, amine, asymmetric/symmetric C-H stretching frequencies of the -CH_2_ group in PEI chains with Si–O and Si–O–Si from sand in the fingerprint region (marked as “c–f”), as well as in the main functional group region (marked as “a,b”).

### Optimization of atrazine degradation parameters

#### UV spectrophotometric analysis

Degradation of atrazine in aqueous solution was studied by following the atrazine UV peak at 222 nm^67^. The solution was treated by adding H_2_O_2_ and one of the following: MK10, MK10_PEI_Cu NPs, sand or sand_PEI_Cu NPs and mixing for 60 min. At the end of the experiments, peaks of atrazine or other degraded products were detected in the aqueous supernatant for treatments with MK10_PEI_Cu NPs and sand_PEI_Cu NPs. To further study the reaction, the solution was collected at the end of the degradation process and extracted with two different eluents: (1) a mixture of methanol-water, and (2) methylene chloride (DCM). A small, broad peak was observed between 235 and 270 nm for both extraction methods, indicating that some low molecular weight metabolites were probably formed during the degradation process (see Fig. S6a in the Supplementary Information). Similarly, atrazine degradation was carried out in the absence of H_2_O_2_ with the modified MK10 and sand materials. The aqueous supernatant did not show any peak. The solid matrix in each case was subjected to elution with DCM and methanol-water mixtures, separately. The extracted liquid phase was subjected to UV spectrophotometric analysis, revealing no extracted atrazine; therefore atrazine degradation was observed only in the presence of H_2_O_2_ and with either modified clay or sand. For the modified MK10 and sand in the absence of H_2_O_2_, atrazine was extracted from the matrix, indicating adsorption and not degradation. A similar procedure was followed to verify the atrazine degradation with unmodified MK10 and sand, with and without H_2_O_2_. The UV-vis spectrophotometric results again showed that the reaction involved adsorption rather than degradation (see Fig. S6a–c in the Supplementary Information). This is discussed further in the section on removal dynamics (Adsorption vs. Degradation).

#### Effect of hydrogen peroxide

**Figure 4:**
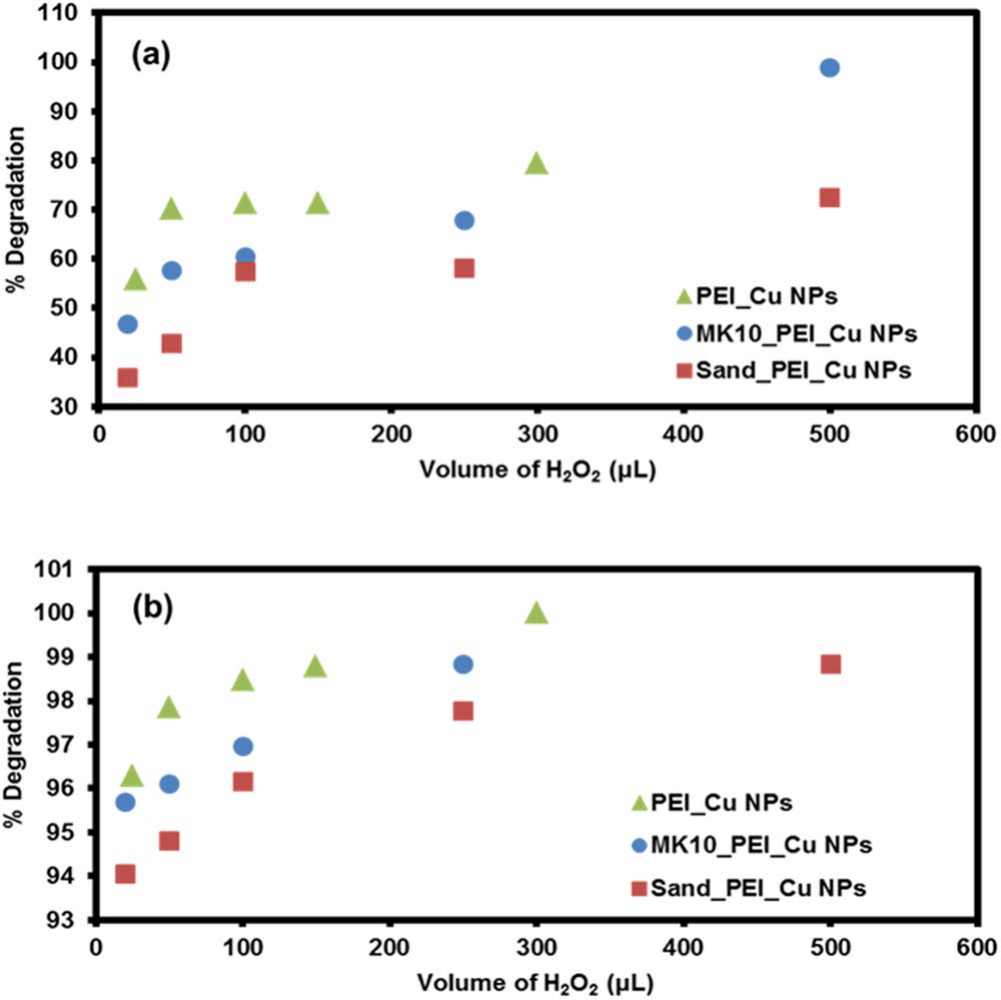
Influence of hydrogen peroxide on degradation of atrazine with modified MK10 and sand at two different equilibrium times: (**a**) 15 h, and (**b**) 1 h.

The effect of hydrogen peroxide (30%) concentration (0.0098–0.245 mole of H_2_O_2_ in 20 mL volume) on atrazine degradation was studied for a PEI_Cu NP suspension (100 µL), and for addition of 10 mg of MK10_PEI_Cu NPs and sand_PEI_Cu NPs. Results are depicted in Fig. 4a,b. The PEI_Cu NP suspension showed maximum degradation (44.8% after 1 h; 77% after 15 h) with addition of 20 μL of hydrogen peroxide (0.0098 M). MK10_PEI_Cu NPs and sand_PEI_Cu NPs showed complete atrazine degradation with 500 μL (0.245 M) of H_2_O_2_ in 1 h (Fig. 4b last points are overlapped). This clearly indicates that the rate of atrazine degradation depends on the copper nanoparticles and their contact time with atrazine. We observed >94% and >35% degradation of atrazine after 15 h and 1 h for all three cases. Furthermore, to reduce the longer kinetics of degradation (15 h, Fig. 4a) and maintain the lower concentration of H_2_O_2_, degradation can be achieved by increasing the number of catalytic reactive sites (i.e., by increasing catalyst concentration as discussed below).

#### Effect of catalyst dosage

**Figure 5:**
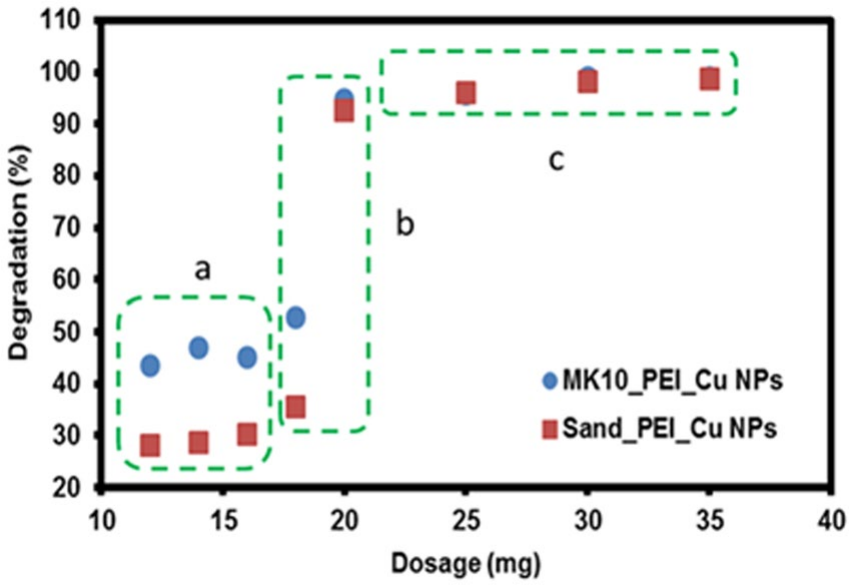
Influence of catalyst dosage on degradation of atrazine. (Conditions: atrazine = 20 mg L^−1^, volume of solution = 20 mL, H_2_O_2_ (30%) = 20 µL); dotted boxes mark different zones of catalyst activity.

Batch experiments were conducted to investigate the effect of dosage of PEI_Cu NPs deposited onto sand and MK10, on atrazine degradation (20 mL of 20 mg L^−1^), by varying dosages in the range of 12–35 mg (which includes the PEI_Cu NPs and MK10/sand); results are shown in Fig. 5. Initially, the degradation in both cases increased slowly by ~10%, and the rate of degradation remained low until 18 mg of the catalyst was added (marked as area “a” by a green dashed box). The lower rate of degradation is possibly due to noncovalent interactions between the catalyst and atrazine, which obstruct contact between H_2_O_2_ and the copper. We observe a sharp increase in the atrazine degradation (to ~95%, marked as area “b” by a green dashed box), when the amount of catalyst increased to 18–20 mg. This is due to increased availability of reactive sites and the existence of more PEI_Cu NPs (which may initiate radical formation), followed by the synergistic influence on the interaction between the catalyst and atrazine (as mentioned earlier). Furthermore, when the amount of added catalyst was raised to 30 mg, there was no significant increase (compared to the 20 mg catalyst dosage) in atrazine degradation (marked as area “c” by a green dashed box in Fig. 5; points are over lapped) consequently, this (30 mg) “saturation dosage” was employed for further study.

#### Effect of pH

**Figure 6:**
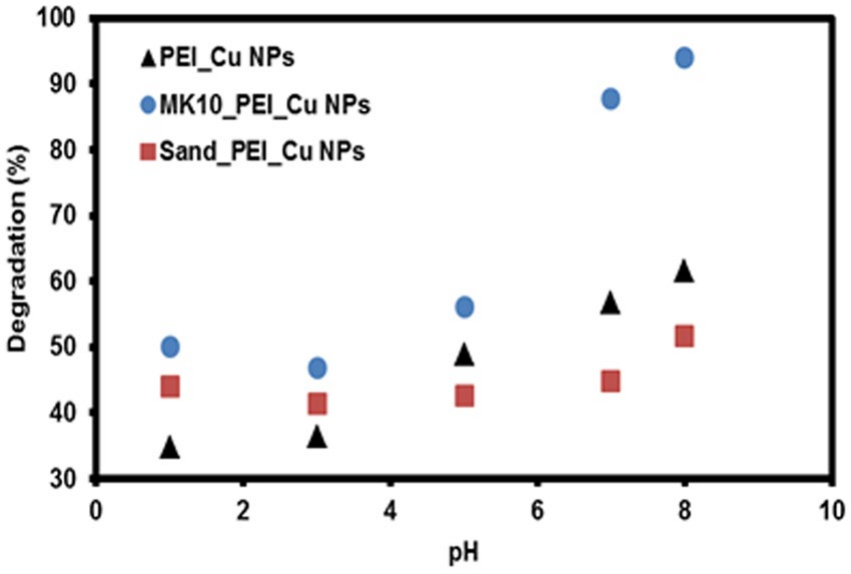
Effect of pH (adjusted with H_3_PO_4_ and K_2_HPO_4_) on degradation of atrazine.

The influence of pH on atrazine degradation with PEI_Cu NPs, modified MK10 and sand was also studied. The pH of the reaction mixture was adjusted with different acids (HCl, H_2_SO_4_ and H_3_PO_4_) and bases (NaOH and K_2_HPO_4_). The pH remained constant throughout the experiments. In all cases, maximum (>99%) degradation of atrazine was observed (this is comparable to regular Fenton type reactions; therefore no plots are shown). Different patterns and levels of atrazine degradation were observed when the solution was treated with H_3_PO_4_ and K_2_HPO_4_ (Fig. 6). This distinct behavior of H_3_PO_4_ and K_2_HPO_4_ adjusted solution pH versus atrazine degradation is discussed hereafter.

The PEI_Cu NP suspension, modified clay and sand show different percentages of atrazine degradation and various patterns (Fig. 6), when plotting atrazine degradation vs. solution pH (adjusted with H_3_PO_4_). The modified MK10 shows a sigmoidal type curve (Fig. 6) and a maximum degradation of 87.8%; modified sand and PEI_Cu NP suspensions show a different degradation pattern with lower degradation rates (44.9% and 63.7%, respectively). The lower atrazine degradation may be influenced by the ionic species of H_3_PO_4_ and its affinity to copper. Furthermore, the steric interference of the aliphatic chains (from PEI) in PEI_Cu NPs cannot be ignored as a factor affecting the degradation.

With this pH condition (stabilized by K_2_HPO_4_), the reaction is faster for alkaline pH (8 > 7 ≫ 6…); this appears to be opposite to the “classical” Fenton reaction, where (strong) acidic conditions are needed. This behavior may be a result of the presence of Cu^2+^, originating from copper (II) phosphate. Here, the copper ion can be released easily for the oxidative degradation reaction.

The structural steric hindrance of atrazine is larger for the carbon next to the ethylamine group, because it does not have an alkyl group that is difficult to break, and it is also one carbon short with respect to the alkylamine (see Fig. S7 in the Supplementary Information)^47^. Therefore, MK10 and sand_PEI_Cu NPs likely degrade the atrazine through one of the following mechanisms: (i) protonation of the amino group, then the aromatic ring, followed by breaking of a C-Cl bond; (ii) direct nucleophilic displacement of Cl by an hydroxyl group, and (iii) a radical mechanism involving replacement of the chlorine atom by an hydroxyl group, followed by reduction of the amino group and oxidation of the alkyl group^67^.

#### Leachability of copper

No UV-vis spectrophotometric peak was observed for PEI-Cu NPs, and ICP measurements detected virtually no copper (less than 0.02% of Cu from the catalyst) in the supernatant after the degradation of atrazine. This confirms that there was no significant leaching of PEI-Cu NPs or copper from the MK10_PEI_Cu NPs and sand_PEI_Cu NPs after the atrazine degradation. The small amount of leachable copper and increased PEI_Cu stability are due to PEI_Cu stoichiometry, in which primary, secondary, and tertiary amines are present; this facilitates additional network integrity, capability and physicochemical support from the host matrices (MK10 and sand) during the degradation.

#### Removal dynamics (Adsorption vs. Degradation)

**Figure 7:**
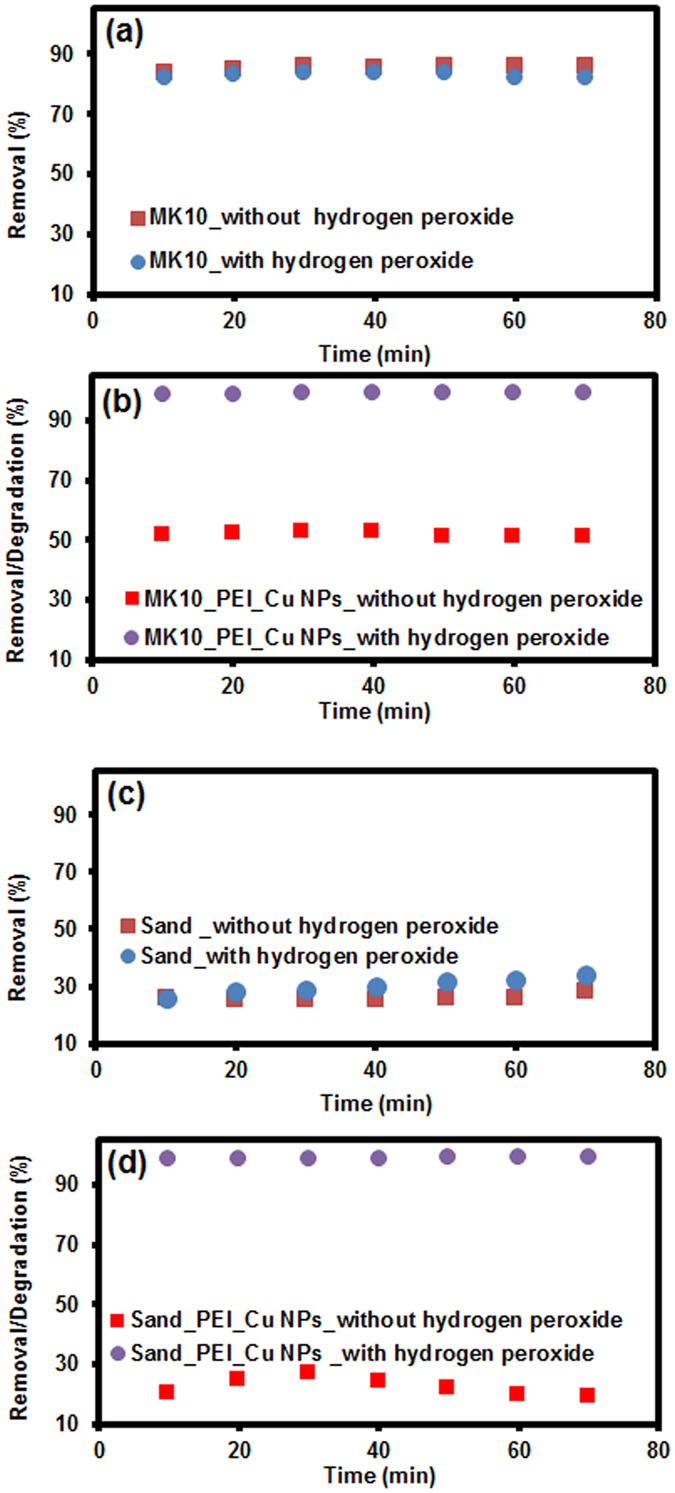
Atrazine degradation dynamics [adsorption/degradation] of (**a**) unmodified MK10, (**b**) modified MK10, (**c**) unmodified sand, and (**d**) modified sand.

To verify the atrazine removal process (adsorption or degradation) by our prepared materials, we followed the same optimum reaction conditions as described above. The results are shown in Fig. 7. The atrazine adsorbed (not degraded) at least partially on both modified and unmodified MK10 and sand surfaces, in the absence of hydrogen peroxide. This was verified by an elution study, as mentioned in the UV spectrophotometric analysis section. For modified MK10 and sand, atrazine molecules can interact relatively strongly with copper, due to the presence of heteroatoms (N) with free electron pairs and aromatic rings with delocalized π electrons^68^. The unmodified MK10 shows only adsorption in the presence and absence of H_2_O_2_. After a reaction time of 60 min, 85.94% and 82.12% of the atrazine was removed from the solution in the presence and absence of H_2_O_2_, respectively (Fig. 7a). It has been noted that atrazine adsorption (not degradation) on unmodified MK10 is significantly higher than its adsorption on modified MK10 (Fig. 7b), in the absence of hydrogen peroxide. This is explained by the dominance of atrazine adsorption through noncovalent interactions on the less crowded MK10 surface^69^. The adsorption may be due to the presence of both Bronsted and Lewis acidic active sites on MK10. The interlayer cations are exchangeable, thus allowing alteration of the acidic nature of the material by simple ion-exchange procedure^70^. A similar pattern of atrazine removal (but with a much lower removal rate) was observed for unmodified sand (34.28% and 28.38%, Fig. 7c,d). Atrazine sorption in all of the above cases was verified by elution experiments. Similarly, the kinetics of atrazine degradation by modified MK10 and sand were also studied in the presence and absence of H_2_O_2_. The results are shown in Fig. 7b and d. When the equilibrium time was increased, the degradation level raised gradually in the presence of H_2_O_2_. Maximum degradation of atrazine for both systems was observed after 60 min, beyond which there was almost no further increase in degradation, for both MK10_PEI_Cu NPs and sand_PEI_Cu NPs; this can define the optimum contact time.

#### Kinetics of degradation

**Figure 8:**
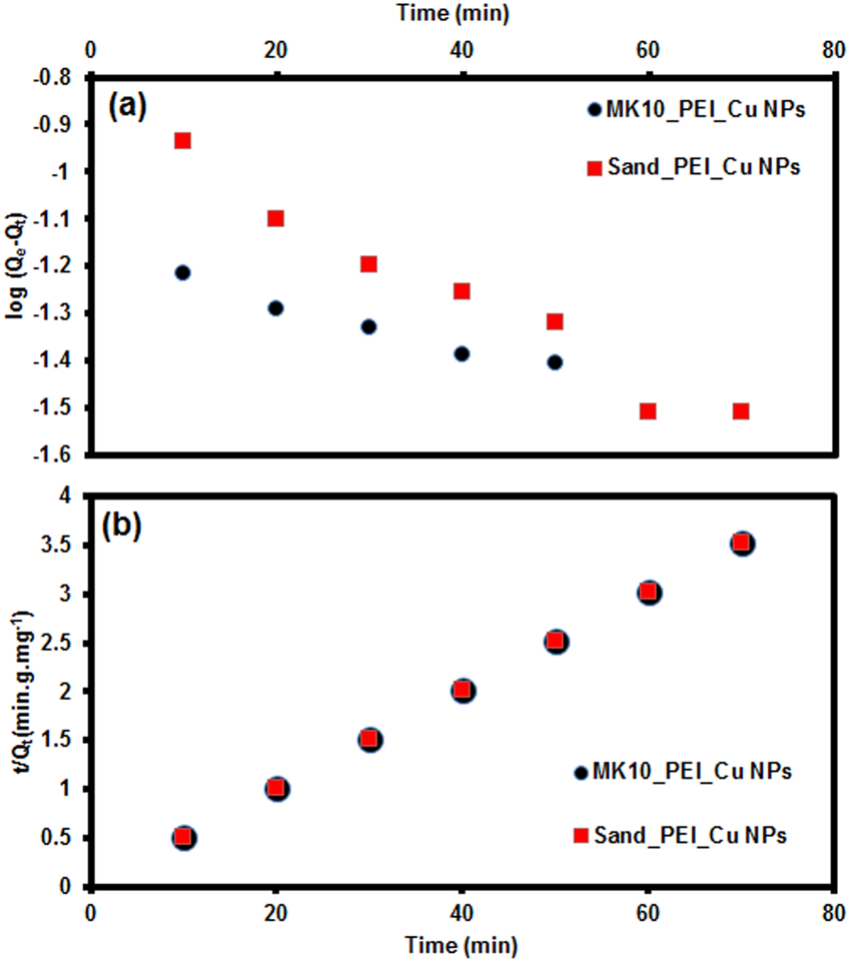
First-order kinetic (**a**) and second-order kinetic (**b**) models of atrazine degradation by modified MK10 and sand.

The degradation of atrazine for all treatments discussed previously is rapid (60 min); this may be related to the affinity of atrazine for the mineral surfaces^68^ at circumneutral pH, followed by its degradation in the presence of H_2_O_2_. The rate of atrazine degradation (20 mg L^−1^) was examined over time (10–70 min at intervals of 10 min). To investigate the degradation mechanism, first-order (equation (1)) and second-order (equation (2)) models^71, 72^ were used to fit the experimental data:

1 $$\mathrm{log}({{\rm{Q}}}_{{\rm{e}}}-{{\rm{Q}}}_{{\rm{t}}})={{\rm{logQ}}}_{{\rm{e}}}-{{\rm{K}}}_{1}\times \frac{{\rm{t}}}{2.303}$$

and

2 $$\frac{{\rm{t}}}{{{\rm{Q}}}_{{\rm{t}}}}=\frac{1}{{{\rm{K}}}_{2}{{\rm{Q}}}_{{\rm{e}}}^{2}}+\{\frac{{\rm{t}}}{{{\rm{Q}}}_{{\rm{e}}}}\}$$

where Q_e_ and Q_t_ (mg g^−1^) are the amounts of atrazine degraded per unit mass of catalyst at equilibrium and time t (min), respectively, and K_1_, and K_2_ are the first- and second-order rate constants. The plots (Fig. 8) of log (Q_e_ − Q_t_) versus time (Fig. 8a) and t/Q_t_ versus time (Fig. 8b) give the kinetic parameters related to first-order and second-order models, respectively. The rate constant K, and Q_e_ (Q_e1_ and Q_e2_ denotes calculated Q_e_ from the first-order and second-order kinetic plots respectively) for MK10_PEI_Cu NPs, were K_1_ = 0.0993 min^−1^, Q_e1_=0.1977 mg g^−1^ [from plot] and r^2^ = 0.7771; and K_2_ = 1.7957 g mg^−1^ min^−1^, Q_e2_ = 24.8757 mg g^−1^ and r^2^ = 0.9999. For sand_PEI_Cu NPs, K_1_ = 0.1168 min^−1^, Q_e1_ = 0.7318 mg g^−1^ and r^2^ = 0.7794; K_2_ = 0.8133 g mg^−1^ min^−1^, Q_e2_ = 24.8756 mg g^−1^and r^2^ = 0.9999. Higher regression coefficients indicate that the degradation data are consistent with the second-order model.

The second-order mechanism indicates that the degradation rate depends not only on the atrazine concentration, but also on additional parameters such as the external surface area of the MK10_PEI_Cu NPs and sand_PEI_Cu NPs, the shape and density of the particles, the concentration of the atrazine, steric hindrance due to bulkier PEI, and the mixing rate. Based on a previously reported atrazine degradation pathway^73^, the following possible mechanism of degradation is suggested. The active sites in MK10_PEI_Cu NPs and sand_PEI_Cu NPs, which contain Cu_2_O and CuO, are recognized as the redox-active species within the layers, pores, and outside surfaces. These Cu NPs show high oxidation activity and are easily reducible, and thus function as reactive centers for oxidative degradation of atrazine and its degradation intermediates in the layers of montmorillonite and micropores of sand in the presence of hydrogen peroxide.

Note that when the experiment proceeds at acidic conditions (pH < 2), the degradation begins with protonation of amino groups preceding electron transfer and de-alkylated products^73, 74^. At alkaline conditions (above pH 8), the degradation starts from substitution of Cl atoms by attack of an anionic hydroxyl group; this may boost the possible production of OH radicals during degradation. In conclusion, at circumneutral pH, oxidation of the alkyl groups of the amines produces a hydroxyl group that leads to degradation^67, 75^.

In all of the above cases, copper is the active site and responsible for the high reactivity attributed to the unique dimeric Cu species (e.g., Cu^2+^-O^2−^-Cu^2+^, Cu^+^-O^2−^-Cu^2+^, and Cu^+^ **···**Cu^2+^-O^76–78^. The entire transition is stabilized by the host (MK10 or sand) and PEI. When Cu^2+^ is the active center, it is anticipated that electrons can be transferred from the oxygen to the metal cations, and the total charge is equilibrated/stabilized from the neighboring amine functional group containing a loan pair of electrons as well as from the host during the degradation. From our previous study, EPR experimental results for the degradation of atrazine with PEI_Cu NPs, the degradation mechanisms involves OH radicals, rather than peroxo radicals, at circumneutral pH^59^.

**Figure 9:**
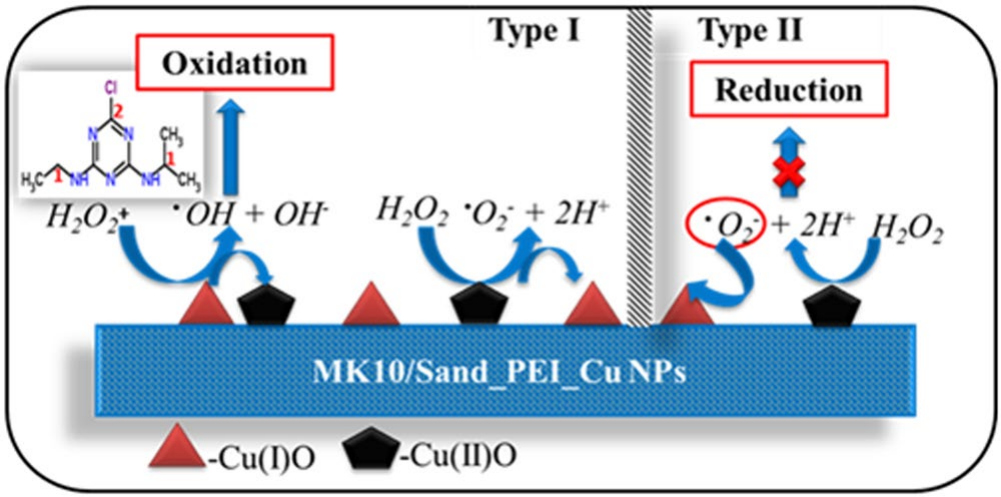
Schematic representation of possible mechanism.

There is no clear picture regarding the nature of the mechanism for transition metals in redox activity, but their reversible reduction and re-oxidation are believed to be crucial. In the current case, as shown in Fig. 9, these activities are possible through stabilization of one or more transition states by the PEI and host matrix for re-oxidation of copper species; this in turn closes the catalytic cycle. MK10 can be used as an efficient and versatile catalyst for various organic reactions such as synthesis of dimethyl acetals, enamines, γ-lactones, enolthioethers, α, β-unsaturated aldehydes and porphyrin synthesis^79^. The interlayer cations are exchangeable, thus allowing alteration of the acidic nature of the material by a simple ion-exchange procedure^70^.

## Summary

A simple preparation of PEI_Cu NPs, with deposition onto MK10 and sand was demonstrated, followed by application to the degradation of atrazine in aqueous solutions. It is concluded that the degradation is due to exclusively the PEI_Cu NPs, which was confirmed by various analytical measurements. Moreover, degradation follows faster kinetics (60 min) with 9.8 mM concentrations of hydrogen peroxide, while the pH remains constant, and degradation remains consistent with different optimized pH conditions. Experimental results suggest that degradation is linked to the interplay among all reagents, and coincides with previously reported degradation mechanisms. Also, the observed COD (chemical oxygen demand) values for atrazine solution before degradation, and after degradation with MK10_PEI_Cu NPs and sand_PEI_Cu NPs, are 170.74, 59.79 and 84.01 mg O_2_/L, respectively. Unmodified MK10 and sand show adsorption (but without degradation) toward atrazine. The above findings suggest that modified MK10 and sand are highly effective materials for advanced oxidation processes, and may improve process performance that can be extended to various pollutants.

## Experimental Section

### Materials

All chemical reagents were used without any purification. Ultrapure water (18.2 MΩ cm^−1^) were used for all experiments. Cupric nitrate trihydrate (CuN_2_O_6_ **·** 3H_2_O, Fluka), polyethylenimine (PEI; H - (NHCH_2_CH_2_)_n_ - NH_2_, branched, M_w_ = 25,000 Da), hydrochloric acid (HCl), phosphoric acid (H_3_PO_4_), methanol, methylene chloride, nitric acid (HNO_3_, >69%), potassium hydrogen phthalate (KHP, Sigma Ultra, minimum 99.95%), AQUANAL™-professional tube test COD (chemical oxygen demand, 0–150 mg L^−1^) and montmorillonite MK10 (H_2_Al_2_(SiO_3_)_4_-nH_2_O; surface area 220–270 m^2^ g^−1^) were obtained from Sigma-Aldrich (Israel); sulphuric acid (H_2_SO_4_), sodium carbonate (Na_2_CO_3_) and dipotassium hydrogen phosphate (K_2_HPO_4_) were purchased from Merck. Sodium hydroxide (NaOH) and Hydrogen peroxide (H_2_O_2_, 30%) were purchased from Bio-lab Ltd., (Israel). Sodium borohydride (NaBH_4_) was purchased from Nile Chemicals (India). Atrazine (99%) – 2-chloro-4-ethylamino-6-isopropylamino-s-triazine (C_8_H_14_ClN_5_) was received from Agan Chemical Manufacturers Ltd. (Israel); sand was obtained from Unimin corporation, USA.

### Characterization of prepared materials

Light absorption spectra (UV-Visible Spectrophotometer, Cary 100 Bio, Varian Inc.) and Zeta-potential (ZP; Zetasizer Nano-ZS, Malvern Instruments) measurements were conducted with diluted PEI_Cu NP suspensions. Dynamic Light Scattering (DLS; Zetasizer Micro V, Malvern) measurements were also carried out with dilute PEI_Cu NP suspensions. Scanning electron microscopy (SEM) images was performed with a Zeiss Supra 55 VP FEG high resolution instrument and the functional group change was recorded using a NICOLET 6700 FT-IR (Thermo Scientific Inc.). All transmittance spectra were measured with KBr as background. Composition of the adsorbent was analyzed using a SDT Q600 V8.3 Build 101 thermal analyzer (DSC-TGA Standard). Samples were heated from room temperature to 750 °C at the heating rate of 20 °C min^−1^ in nitrogen atmosphere in an alumina pan. XRD patterns were obtained on an Ultima III (Rigaku, Japan) model powder diffractometer using Cu Kα radiation with 2θ degree scan (Bragg-Brentano mode), 40 kV and 40 mA. XPS measurements were carried out with a Kratos AXIS ULTRA system using a monochromatic Al Kα X-ray source (hν = 1486.6 eV) at 75 W and detection pass energies ranging between 20 and 80 eV. A low-energy electron flood gun (eFG) was applied for charge neutralization. To define binding energies (BE) of different elements, C 1 s line at 284.8 eV 1,3 was taken as a reference. Curve fitting analysis was based on Shirley background subtraction and application of Gaussian-Lorenzian line shapes^60–62^. LabRAM HR Evolution (Horiba, France) using two laser lines (532 nm 2 mW maximum power on the sample, and 785 nm 8 mW maximum power on the sample) allowing for Raman measurements from 50 cm^−1^ and onward. The instrument is equipped with an 850 mm spectrograph. The system spectral resolution is better than 10 cm^−1^ when working with the 300 g/mm that was used in these measurements. The sample is illuminated using ×100 objective (MPlanFL N NA 0.9, Olympus, Japan). The LabRAM measured using a 1024 × 256 pixel Open Electrode front illuminated CCD camera cooled to −60 °C (Syncerity, Horiba, USA). The system uses an open confocal microscope (Olympus BXFM) with spatial resolution better than 1 μm.

### Synthesis of PEI_Cu NPs

Stock solution of 1.6 mM PEI in ultra-pure water was prepared. 4 mL of the PEI stock solution were then mixed for 5 min with 5 mL of 250 mM Cu(NO_3_)_2_ · 3H_2_O solution and ultrapure water were added to achieve total volumes of 40 mL. Subsequently, 10 mL of 0.5 M NaBH_4_ was added into the solution to reduce the copper cation to copper oxide^59^.

The 50 mL Cu-NP suspensions were stirred (~350 rpm) for 1 h and then dialyzed for 1 day (Cellu Sep: 3500 MWCO, Membrane Filtration Products, Inc., TX, USA) in glass beakers filled with 950 mL DI water. The final PEI_Cu NPs suspension was monodisperse with average particle size of 55.33 nm and zeta potential of 33.4 mV. Further studies were carried out with the above PEI_Cu NP suspension.

### Preparation of PEI_Cu NPs deposited onto MK10 and sand

Known amounts of sand and MK10 were activated in an oven for 2 h at 150 °C and stored in glass vials for further use. Known amounts of the activated sand and MK10 (5 g) were sonicated with 20 mL of PEI_Cu_NP solution followed by 12 h stirring. The product was then filtered, washed with excess of water until the supernatant pH was neutral, and dried in an oven at 60 °C. A known weight of the prepared materials (before and after the atrazine degradation) was treated with acidic water (0.1% HNO_3_) to quantify the concentrations of copper using an inductive coupled plasma mass-spectrophotometer (ICP-MS, Agilent 7700).

### Degradation experiment

We applied a similar degradation procedure as in our previous publication^59^. A known amount of each of MK10_PEI_Cu NPs and sand_PEI_Cu NPs was stirred with 20 mg L^−1^ of atrazine, 20 mL of H_2_O_2_ (30%) at pH range of 6–7 and the total volume of 20 mL. The entire reaction mixture was stirred at 500 rpm for 1 h under open atmospheric conditions. The kinetics of the reaction were measured at each predetermined time interval, 1 mL of the solution was filtered through a 0.22 µm microfiltration disk (PVDF-0.22 µm, Millex-GV, Millipore) and 25 µL of the filtrated solution was injected to high pressure liquid chromatograph (HPLC; 1525 Binary HPLC Pump, Waters) with UV detector (2487 Dual λ Absorber Detector, Waters) measured at λ = 222 nm. Eluent (75% acetonitrile: 25% deionized water) flow rate was 1 mL min^−1^ with pressure of ~1500 psi. The various analytical parameters were optimized by examining different concentrations of H_2_O_2_, catalyst dosage, and concentration of atrazine. The solution pH was monitored at the end of each experiment. Also, in other catalytic experiments, the same procedure was followed for the unmodified MK10, sand and PEI_Cu NP suspension.

COD experiments were carried out with AQUANAL™-professional tube test COD (0–150 mg L^−1^). For experiments in the COD test tube, a known volume of supernatant after the atrazine degradation was added to 1 mL of Na_2_CO_3_ (50 mg L^−1^) and the entire reaction mixture was refluxed at 150 °C for 2 h, followed by cooling and subjected to UV-vis spectroscopic analysis. The samples were covered to minimize evaporation losses and heated in water. Sodium carbonate was used to prevent the peroxide from interfering in measurement of COD^80^. Earlier, calibration was done with KHP as above in the concentration range of 15 to 125 mg L^−1^.

## Electronic supplementary material

Supplementary information
