# Supplementary material for: Atrazine degradation through PEI-copper nanoparticles deposited onto montmorillonite and sand
Source: Sci Rep. 2017 May 3;7:1415. doi: 10.1038/s41598-017-01429-5 (PMC5431096; doi:10.1038/s41598-017-01429-5)
Supplement: Supplementary file 1 — Supplementary information [file 41598_2017_1429_MOESM1_ESM.pdf]

Supplementary Information

Atrazine degradation through PEI-copper nanoparticles deposited onto montmorillonite and sand

Sethu Kalidhasan[1,*], Ishai Dror[1, †], and Brian Berkowitz[1, †]

[1]Department of Earth and Planetary Sciences, Weizmann Institute of Science, Rehovot 7610001, Israel

[*]Corresponding author E-mail: sethu.kalidhasan@weizmann.ac.il; skalidasan@gmail.com

[†] These authors contributed equally to this work

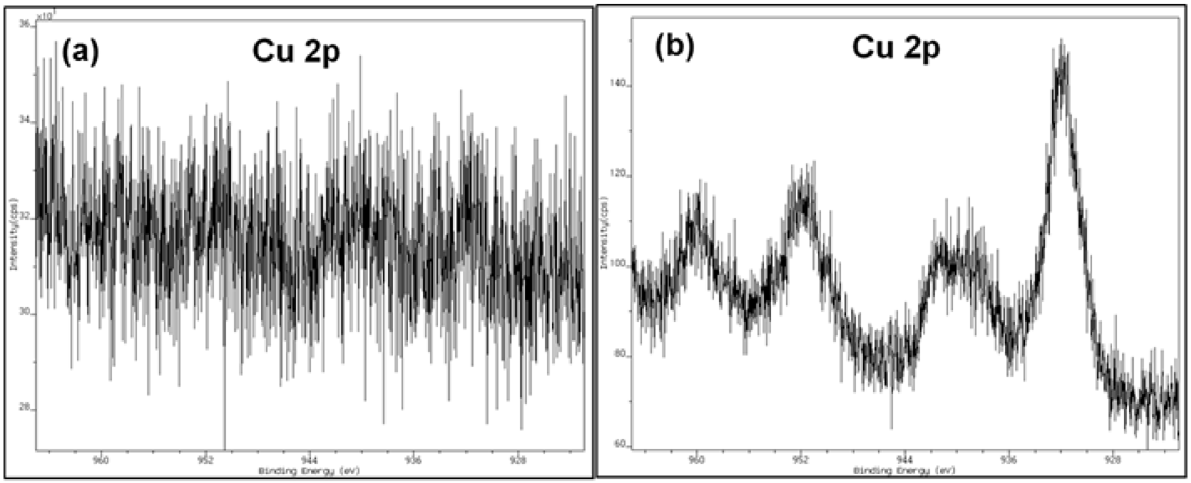

(c)

Peak	Type	Position BE (eV)	FWHM (eV)	Raw Area (cps eV)	RSF	Atomic Mass	Atomic Conc %	Mass Conc %
O 1s	Reg	530.525	1.924	9842.0	0.780	15.999	62.50	50.53
Al 2p	Reg	72.625	1.022	238.7	0.193	26.982	6.72	9.16
Si 2p	Reg	101.425	1.802	1464.7	0.328	28.086	24.10	34.20
Cu 2p-3/2	Reg	940.125	0.038	229.8	5.321	63.549	0.20	0.63
Cu 2p-1/2	Reg	950.300	0.102	157.3	5.321	63.549	0.13	0.43
N 1s	Reg	400.075	0.154	91.6	0.477	14.007	0.98	0.69
C 1s	Reg	282.900	0.411	225.6	0.278	12.011	4.26	2.59
K 2p	Reg	291.450	0.100	71.1	1.466	39.102	0.25	0.50
Mg 1s	Reg	1301.775	0.503	406.6	2.260	24.312	0.59	0.73
Ca 2p	Reg	350.550	0.102	95.8	1.833	40.078	0.27	0.55

(d)

Peak	Type	Position BE (eV)	FWHM (eV)	Raw Area (cps eV)	RSF	Atomic Mass	Atomic Conc %	Mass Conc %
O 1s	Reg	529.000	1.798	7331.0	0.780	15.999	51.30	41.41
Al 2p	Reg	71.825	0.127	42.6	0.193	26.982	1.32	1.80
Si 2p	Reg	99.525	1.621	1173.1	0.328	28.086	21.27	30.13
Mg 1s	Reg	1300.050	0.051	245.7	2.260	24.312	0.39	0.48
Ca 2p	Reg	347.775	0.038	49.5	1.833	40.078	0.15	0.31
Cu 2p-3/2	Reg	931.900	2.666	2449.9	5.321	63.549	2.30	7.38
Cu 2p-1/2	Reg	951.275	1.864	1258.9	5.321	63.549	1.18	3.77
N 1s	Reg	397.350	1.542	405.1	0.477	14.007	4.78	3.38
C 1s	Reg	283.300	2.060	801.5	0.278	12.011	16.68	10.10
K 2p	Reg	291.000	1.033	161.0	1.466	39.102	0.63	1.25

Figure S1. XPS pattern of (a) MK10_PEI_Cu NPs, (b) sand_PEI_Cu NPs, and quantification report of (c) MK10_PEI_Cu NPs, and (d) sand_PEI_Cu NPs. The MK10_PEI_Cu NPs sample shows weaker peak signals for copper, due to the presence of the non-conducting PEI - polymeric alkyl chain which coats the copper particles; the weaker peak signals are due also to the lower atomic abundance of copper in the sample (0.2 and 0.13%, see Fig. S1C). To overcome this limitation, we used a charge neutralizer during measurements. As a result, all peaks are shifted slightly to lower binding energy (BE) values, compared to the literature.[1-3] The shift in BE was found to be 1.9 eV for MK10_PEI_Cu NPs and 1.7 eV for sand_PEI_Cu NPs. In addition, use of a charge neutralizer during measurements may change the oxidation state of copper, driving the copper to its higher oxidation state (+2).

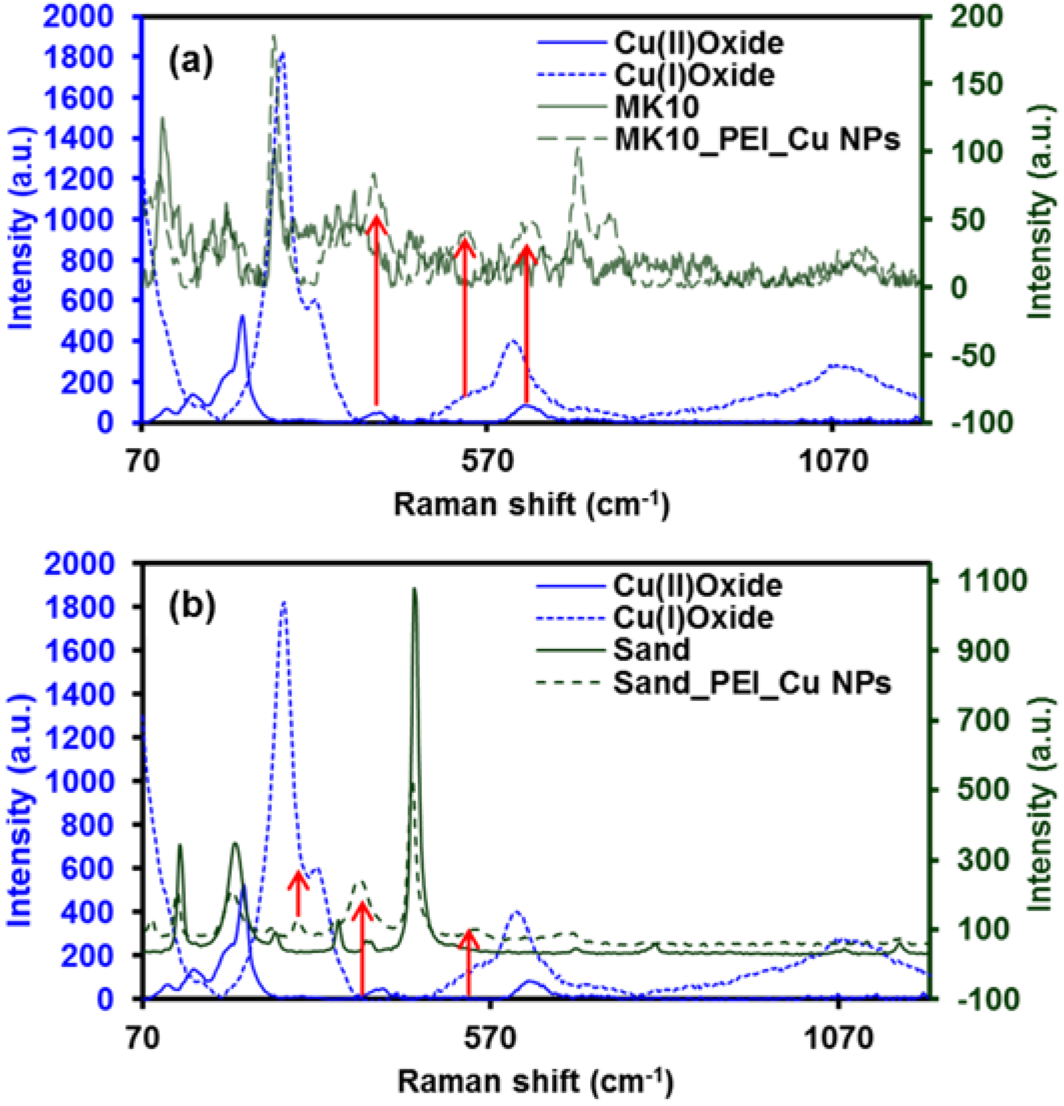

Figure S2. Raman spectrum of (a) clay and (b) sand before and after deposition of PEI_Cu NPs. Red arrows point to changes in the Raman spectrum induced by the presence of Cu (I) and Cu (II) oxide following PEI_Cu NP deposition.

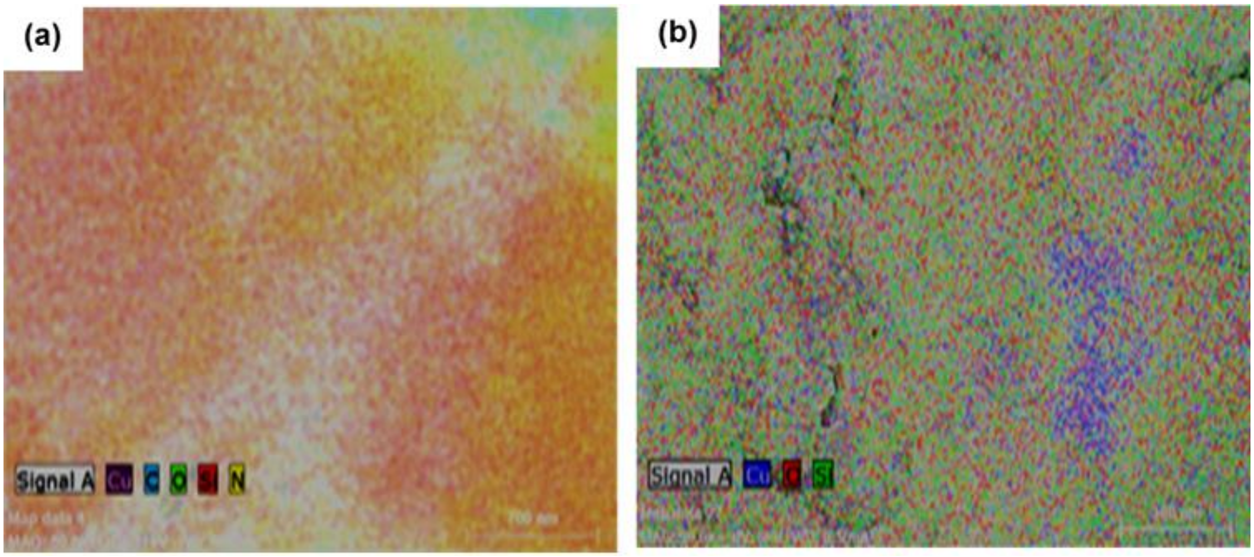

Figure S3. Elemental mapping of copper on (a) MK10_PEI_Cu NPs, and (b) sand_PEI_Cu NPs.

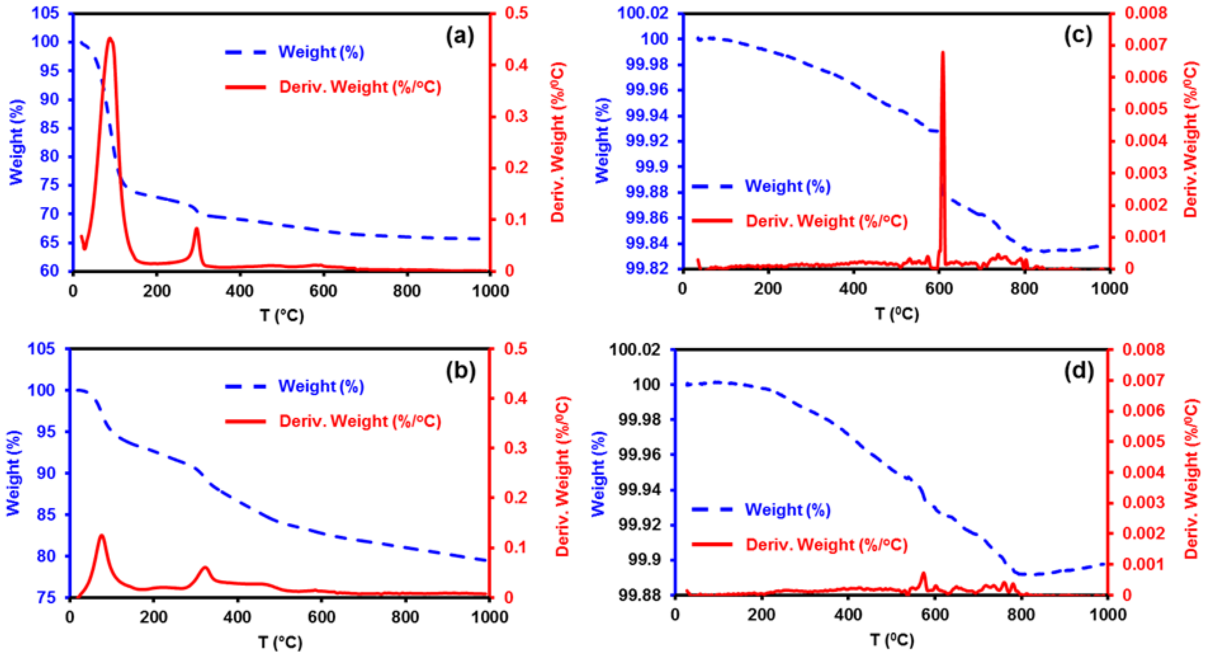

Figure S4. Thermogravimetric analysis of (a) unmodified MK10, (b) MK10_PEI_Cu NPs, (c) unmodified sand, and (d) sand_PEI_Cu NPs.

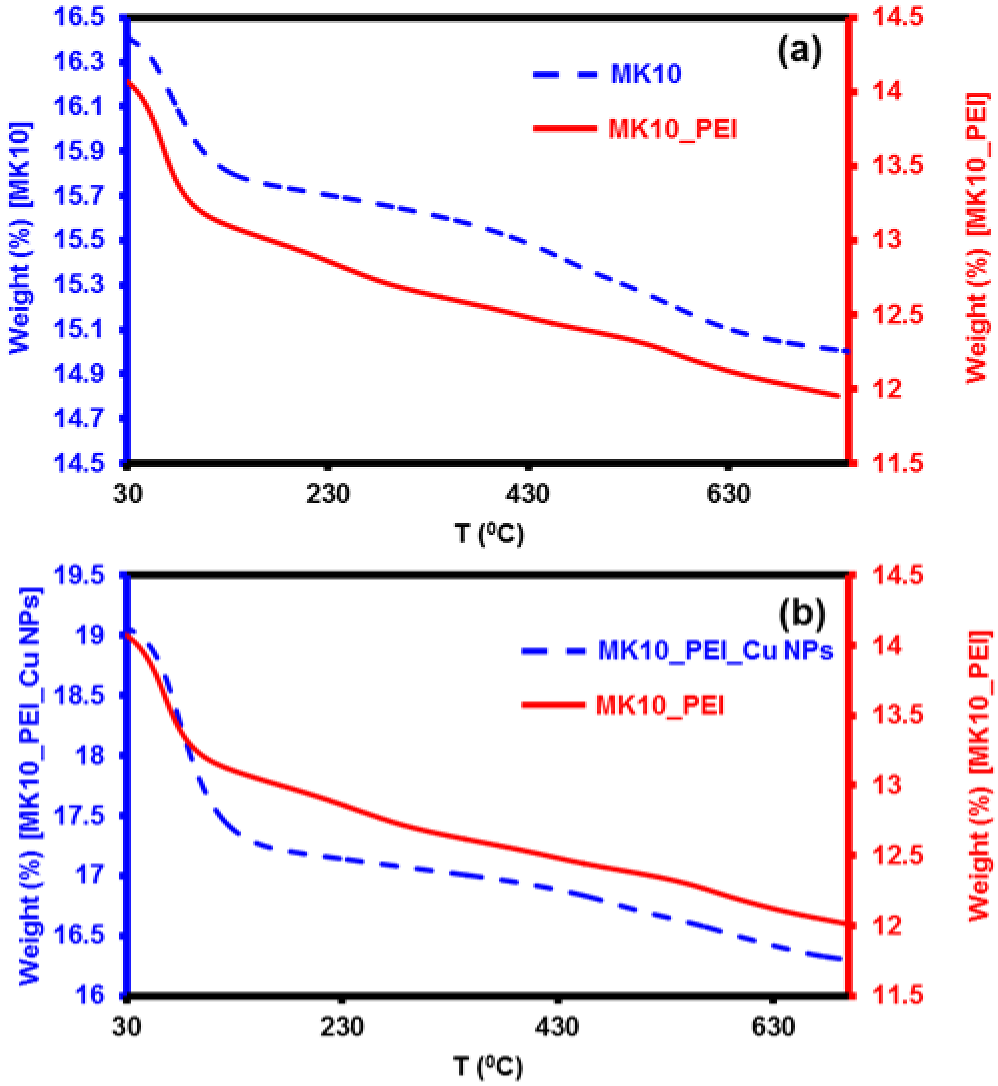

Figure S5. Thermogravimetric analysis of unmodified and modified (a) MK10 vs MK10_PEI, and (b) MK10_PEI vs MK10_PEI Cu NPs.

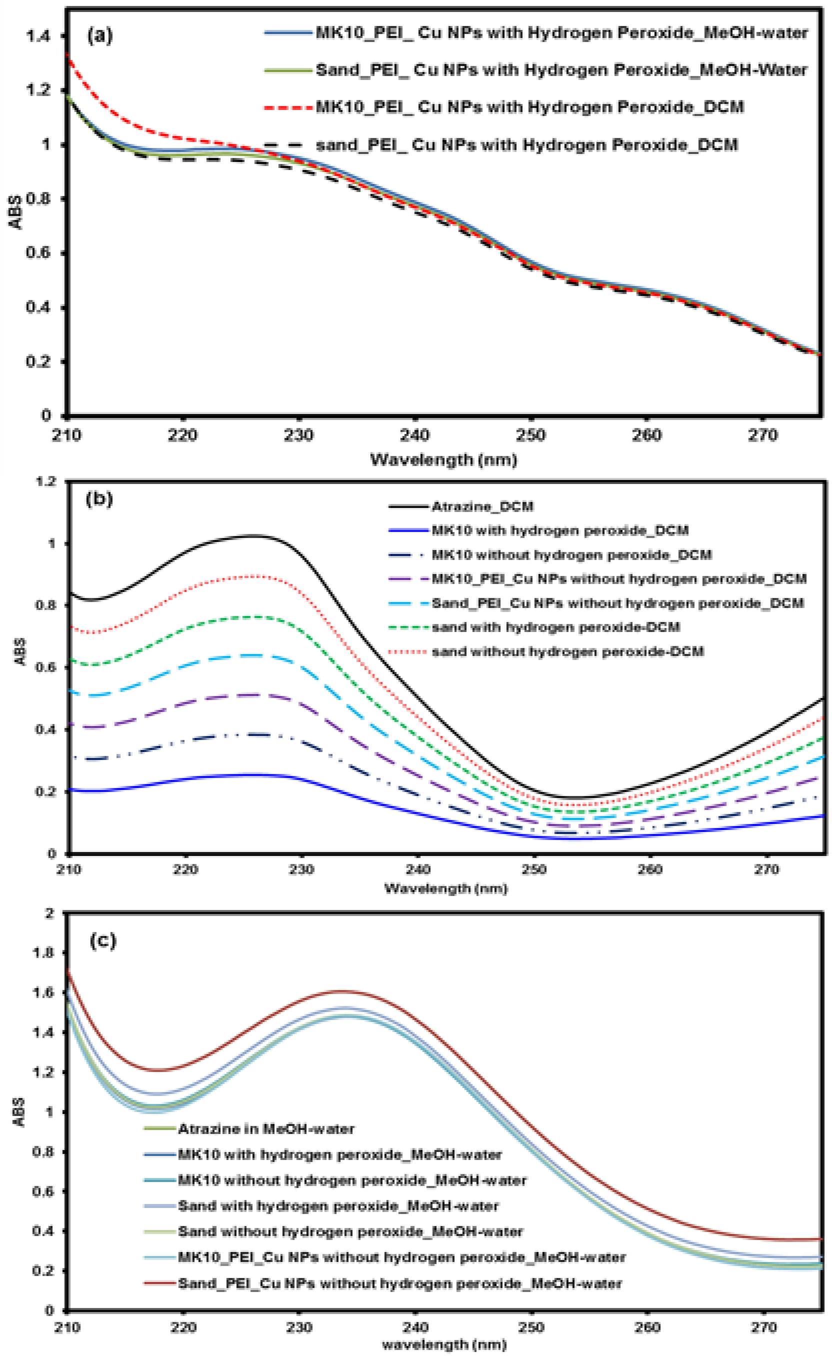

Figure S6. UV-vis spectrum of dynamic of atrazine degradation: (a) Atrazine degradation with PEI_Cu NPs deposited clay and sand in the presence of hydrogen peroxide after eluting with DCM and methanol-water separately; Unmodified materials with and without hydrogen peroxide (modified material without hydrogen peroxide is included in this) eluting with (b) DCM, and (c) methanol-water.

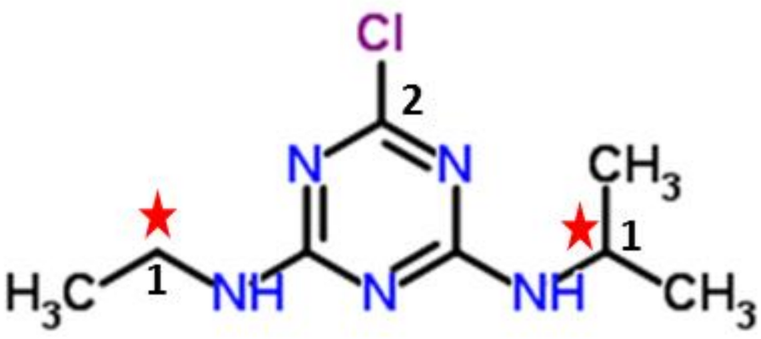

Figure S7. Structure of atrazine, with red stars marking possible bond breaking positions.

Table 1S. The possible peak positions for PEI_Cu NPs deposited on MK10 and sand.

**Wave number (cm^{-1})**	**Description**	**Spot**
3300–3630	Ionic bonded N–H stretching and O–H stretching of structural hydroxyl group from clay	a
2850-2960	C–H asymmetric stretching	b
1650	O–H deformation of entrapped water in clay and N–H bending	c
950-1450	Si–OH vibration, Si–O in-plane stretching, CH_3 rocking, overlap of C–C stretching, CH_2 twisting, C–N stretching, CH_2 rocking and skeletonic stretching	d
820-920	Al–Al-OH deformation, C–H bending out of plane and C–C skeletonic stretching	e
400-800	Si–O stretching of quartz and silica, Si–O deformation perpendicular to optical axis, Si–O deformation parallel to optical axis, Si–O-Si deformation, CH_2 rocking, N–H out of plane wagging, C–C bending and copper oxide.<br>***NOTE:*** Coupled Al–O and Al–O-Si deformation are found only in unmodified and modified MK10.	f

References

[1] Beamson, G. & Briggs D. High resolution XPS of organic polymers: the Scienta ESCA 300 database. John Wiley and Sons; (1992).

[2] Moulder, J. F., Stickle, W. F., Sobol, P. E. & Bomben, K. D. Handbook of X-ray Photoelectron Spectroscopy. Perkin-Elmer Corporation; (1992).

[3] Naumkin, A. V., Kraut-Vass, A., Gaarenstroom, S. W. & Powell, C. J. NIST X-ray Photoelectron Spectroscopy Database, Version 4.1; (2012).
